# *Smyd2* is a Myc-regulated gene critical for MLL-AF9 induced leukemogenesis

**DOI:** 10.18632/oncotarget.12012

**Published:** 2016-09-13

**Authors:** Sevgi Bagislar, Arianna Sabò, Theresia R. Kress, Mirko Doni, Paola Nicoli, Stefano Campaner, Bruno Amati

**Affiliations:** ^1^ Department of Experimental Oncology, European Institute of Oncology, Milan, Italy; ^2^ Center for Genomic Science of IIT at SEMM, Fondazione Istituto Italiano di Tecnologia, Milan, Italy

**Keywords:** MLL-AF9, acute myeloid leukemia, Smyd2, c-Myc, lymphoma

## Abstract

The Smyd2 protein (Set- and Mynd domain containing protein 2) is a methyl-transferase that can modify both histones and cytoplasmic proteins. Smyd2 is over-expressed in several cancer types and was shown to be limiting for tumor development in the pancreas. However, genetic evidence for a role of Smyd2 in other cancers or in mouse development was missing to date. Using germ line-deleted mouse strains, we now show that Smyd2 and the related protein Smyd3 are dispensable for normal development. Ablation of Smyd2 did not affect hematopoiesis, but retarded the development of leukemia promoted by MLL-AF9, a fusion oncogene associated with acute myeloid leukemia (AML) in humans. Smyd2-deleted leukemic cells showed a competitive disadvantage relative to wild-type cells, either *in vitro* or *in vivo*. The *Smyd2* gene was directly activated by the oncogenic transcription factor Myc in either MLL9-AF9-induced leukemias, Myc-induced lymphomas, or fibroblasts. However, unlike leukemias, the development of lymphomas was not dependent upon Smyd2. Our data indicate that Smyd2 has a critical role downstream of Myc in AML.

## INTRODUCTION

Translocations involving MLL are associated with poor prognosis in AML. The MLL-AF9 rearrangement, in particular, was found in 30.4% of all AML patients [1]. The abnormal transcriptional program imposed by the MLL-AF9 fusion protein causes a blockade to myeloid differentiation and maintains a state of self-renewal that is similar to that of embryonic stem cells [2]. In recent years, several epigenetic regulators have emerged that play critical roles in MLL-induced leukemia, such as the methyltransferase Dot1L, the Polycomb repressive complex 2 (PRC2) or the chromatin remodeling complex SWI/SNF [3–6]. In a study that characterized the cellular programs underlying oncogene addiction in AML, MLL-AF9 was shown to be required for expression of the *Smyd2* gene, as well as of the oncogenic transcription factors Myb and Myc, albeit the mechanisms underlying *Smyd2* regulation remained unclear [7]. As will be shown in more detail in this work, genome-wide datasets produced in our laboratory [8, 9] pointed to a direct regulation of *Smyd2* by Myc. We thus decided to address the role of *Smyd2* in MLL-AF9- and Myc-induced malignancies.

Over 50 human genes encode SET-domain methyltransferases: five of these cluster into the Smyd subfamily, in which the SET domain is split by an intervening MYND domain that can mediate protein-protein interactions [10, 11]. Three family members, Smyd1, -2 and -3, share a high degree of sequence homology and were proposed to control gene expression through histone methylation [12–14]. Smyd-family members have been involved both in development and cancer. Deletion of the *Smyd1* (or *Bop*) gene resulted in defective cardiac maturation and embryonic lethality [11]. Smyd3 is over-expressed in hepatocellular and colorectal carcinomas [12, 15]; recent work showed that it is required for the development of those tumors through the up-regulation of a set of cancer promoting genes [16], and also enhances the tumorigenic capacity of esophageal squamous cell carcinoma [17].

Smyd2, the focus of the present work, is expressed in embryos and in a wide range of normal tissues [14]. The human *SMYD2* gene maps to the chromosomal region 1q32, which is amplified in diverse human solid tumors. Its over-expression was associated with poor prognosis in esophageal squamous cell carcinoma (ESCC) [18, 19], childhood acute lymphoblastic leukemia (ALL) [20] and gastric cancer [21]. Recent studies showed that Smyd2 over-expression may be critical in different tumor types, including HPV-unrelated head-and-neck carcinoma [22], pancreatic ductal adenocarcinoma (PDAC) [23], as well as CLL, where together with SMYD3 it may be associated with the acquisition of complex karyotypic alterations [24]. In a mouse model of PDAC, in particular, genetic ablation of *Smyd2* significantly delayed tumor progression [23].

The involvement of Smyd2 in gene regulation *via* histone methylation remains unclear. Smyd2 was first proposed to methylate H3 Lys36 and to associate with the Sin3A histone deacetylase complex to repress gene expression [14]. A subsequent study reported that H3 Lys4 methylation by Smyd2 correlated with up-regulation of a set of genes [25]. On the other hand, several reports indicated that SMYD2 methylates a series of non-histone proteins that may also impact gene expression. First, SMYD2 was reported to methylate p53 on lysine 370, repressing its activity [26]. Biochemical characterization revealed that SMYD2 preferentially binds and methylates p53 rather than histones *in-vitro* [27, 28]. Another non-histone substrate is the tumor suppressor RB, which can be methylated by SMYD2 at lysine 860, an event regulated both through the cell cycle and in response to DNA damage [29]. SMYD2 also methylates RB on lysine 810, leading to increased serine 807/811 phosphorylation and release of the E2F transcription factor, thus favoring E2F activity and cell growth [30]. Hence, SMYD2 appears to antagonize both of the major tumor suppressors, p53 and RB. SMYD2 also methylates the estrogen receptor α (ERα), antagonizing its function as a transactivator [31], as well as PARP1, favoring its poly(ADP-ribosyl)ation activity [32].

The substrate specificity of Smyd2 might be more complex than anticipated [33] and might extend beyond nuclear activities, as SMYD2 appears to lack a nuclear localization signal (NLS) and predominantly localizes to the cytoplasm [34]. One of the cytoplasmic substrates of SMYD2 is Hsp90, methylation of which may play a significant role in muscle myofilament organization [34]. It is noteworthy here that, unlike Smyd1, Smyd2 has not been found to play a role in cardiac development in the mouse [35, 36]. In PDAC, finally, Smyd2 was proposed to coordinate growth and stress signals in part through the methylation of the protein kinase MAPKAPK3 [23].

In this study, we report that germ-line deletion of Smyd2 has no impact on normal embryonic development. Smyd2 knockout mice were born healthy, grew to adulthood with no observable defects, and showed a lifespan comparable to that of control animals. Moreover, combined loss of Smyd2 and of the closest family member, Smyd3, had no effect on survival. Our analyses showed that normal hematopoiesis was not significantly affected by Smyd2 loss. On the other hand, Smyd2 deletion from HSCs significantly delayed the progression of MLL-AF9 induced leukemia, Smyd2-deleted leukemic cells showing a substantial competitive disadvantage relative to control cells. Finally, our data indicated that Smyd2 expression is controlled by Myc. However, despite the prominent phenotype observed in AML, Smyd2 deletion did not affect Myc-induced lymphomagenesis. We propose that Smyd2 may have a specific role in a Myc-dependent leukemogenesis program.

## RESULTS

### Smyd2 knockout and Smyd2; Smyd3 double-knockout mice are viable and healthy

In order to assess the physiological importance of mammalian Smyd2 in survival and development, we used mice carrying a conditional knockout allele (*Smyd2^lox^*) and derived germ-line deleted *Smyd2*^+/−^ animals (see Methods), which were intercrossed to generate *Smyd2*^−/−^ mice. *Smyd2*^−/−^ mice were viable and born at Mendelian frequency, with 99 live-born pups yielding 28 *Smyd2*^+/+^, 41 *Smyd2*^+/−^, and 30 *Smyd2*^−/−^ animals. Over an 18-month observation period, *Smyd2*^−/−^ mice showed a lifespan comparable to that of heterozygous and wild-type controls, were fertile (Supplementary Table 1) and showed no obvious tumor predisposition. Thus, *Smyd2* is a non-essential gene in the mouse.

**Figure 1:**
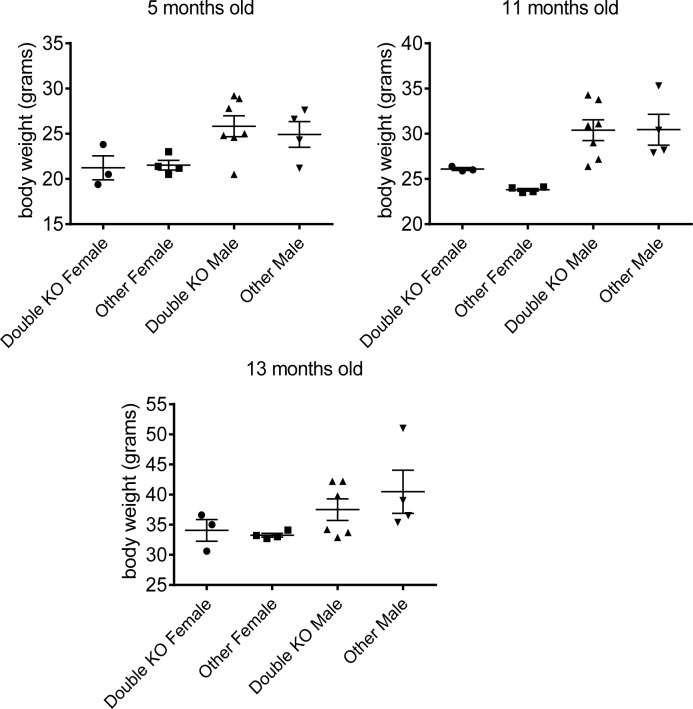
Smyd2/Smyd3 double knock-out does not affect body weight Weight of male and female mice of different genotypes at the indicated ages (5, 11, and 13 months old). The mice studied here were born from breedings between *Smyd2*^+/−^*; Smyd3*^+/−^ and *Smyd2*^+/−^*; Smyd3*^+/−^ parents. Double-deleted mice lacking both *Smyd2* and *Smyd3* are compared with littermates of all other genotypes.

Comparison of protein sequences among Smyd family members showed that Smyd2 and Smyd3 are the closest members of the family (Table 1). We hypothesized that these proteins may functionally compensate each other, thereby masking their potential roles in survival and development. In order to test this hypothesis, we derived *Smyd3* knockout mice (see Methods) and crossed the *Smyd2* and *Smyd3* mutant strains (Table 2): accounting for stochastic variation, the data showed that single and double-mutant mice were born at near-Mendelian ratios, reached adulthood without evident pathologies, were fertile, and showed weights and life-spans comparable to those of control littermates (Figure 1, Supplementary Table 1). Altogether, our data show that *Smyd2* and *Smyd3* are dispensable for development and survival.

**Table 1:** Percent identities of the amino acid sequences of Smyd1, 3, 4 and 5 relative to Smyd2

	Smyd1	Smyd3	Smyd4	Smyd5
**Full-length**	26.3	30.4	11.9	11.2
**SET-domain**	32.5	33	21.9	12.3
**MYND-domain**	56.4	46	35	30

**Table 2:** Breeding strategy for the derivation of Smyd2/3 double knockout mice and observed frequencies of each genotype

i) Breeding: *Smyd2*^+/−^; *Smyd3*^+/−^ with *Smyd2*^+/−^; *Smyd3*^−/−^ mice		
Genotype of pups (*Smyd2*; *Smyd3*)	Observed Frequency (nr.)	Expected Frequency (nr.)
+/+ +/−	0% (0)	12.5% (2)
+/+ −/−	31.25% (5)	12.5% (2)
+/− +/−	25% (4)	25% (4)
+/− −/−	12.5% (2)	25% (4)
−/− +/−	25% (4)	12.5% (2)
−/− −/−	6.25% (1)	12.5% (2)
Total	100% (16)	

ii) Breeding: *Smyd2*^+/−^; *Smyd3*^−/−^ with *Smyd2*^−/−^; *Smyd3*^+/−^ mice		
Genotype of pups (*Smyd2/Smyd3*)	Observed Frequency (nr.)	Expected Frequency (nr.)
+/− +/−	34.78 % (24)	25% (17-18)
+/− −/−	20.28 % (14)	25% (17-18)
−/− +/−	26.08% (18)	25% (17-18)
−/− −/−	18.84% (13)	25% (17-18)
Total	100% (69)	

iii) Breeding: *Smyd2*^−/−^; *Smyd3*^−/+^ with *Smyd2*^−/−^; *Smyd3*^−/+^ mice		
Genotype of pups (*Smyd2/Smyd3*)	Observed Frequency (nr.)	Expected Frequency (nr.)
−/− +/+	15.625% (5)	25% (8)
−/− +/−	56.25% (18)	50% (16)
−/− −/−	28.125% (9)	25% (8)
Total	100% (32)	

### *Smyd2* deletion has no effect on blood or bone marrow cell numbers

**Figure 2:**
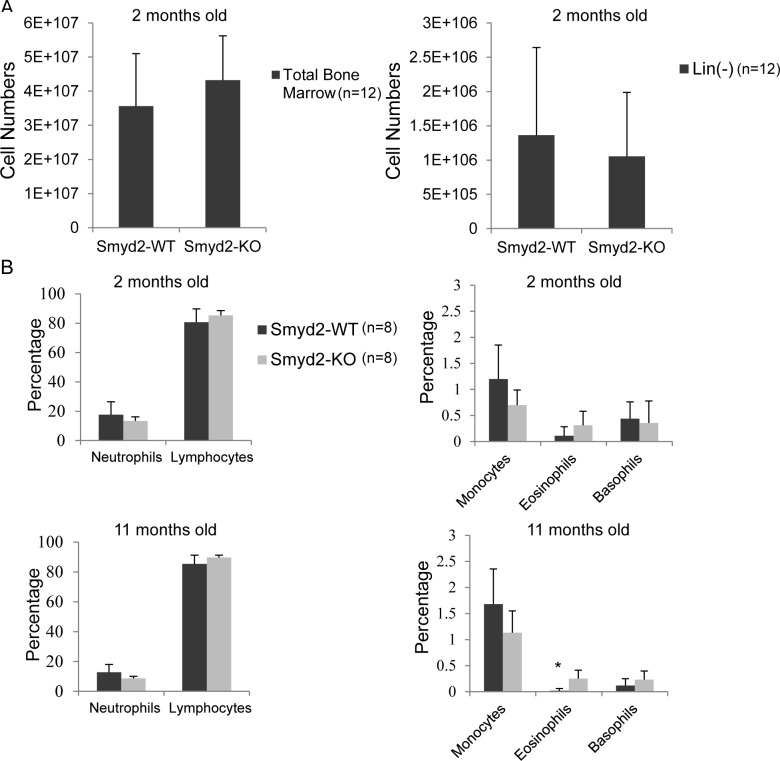
Smyd2 deletion affects neither bone marrow, nor peripheral blood cell counts **A.** Total and Lineage-depleted bone marrow cell counts in *Smyd2*^+/+^ and *Smyd2*^−/−^ mice (*n* = 12 each). P-values are 0.2 and 0.5, respectively. **B.** Peripheral blood samples were collected from the tail vein of 8 *Smyd2*^+/+^ and 8 *Smyd2*^−/−^ mice at 2 and 11 months of age, as indicated, and directly analyzed in an automated blood cell counter (AcT 5 diff, Beckman Coulter) to determine the numbers of neutrophils, lymphocytes, monocytes, eosinophils and basophils. P-values for the difference in counts between WT and KO mice were 0.23, 0.22, 0.08, 0.1 and 0.68 respectively at 2 months, and 0.17, 0.18, 0.16, 0.02 (marked by the asterisk) and 0.23 respectively at 11 months (calculated using 2-tailed Student's *t*-test).

As a premise to address the effect of *Smyd2* deficiency in AML, we first assessed its effect on hematopoiesis. No significant differences were observed in the numbers of either total or Lineage-negative (Lin^−^) cells in the bone marrow of *Smyd2*^−/−^ relative to wild type mice (Figure 2A). Peripheral blood analysis revealed no significant difference between the percentages of mature blood cell populations (neutrophils, lymphocytes, eosinophils and basophils) in *Smyd2*^−/−^ and *Smyd2*^+/+^ mice, at either 2 or 11 months of age (Figure 2B). We noted a slight increase in eosinophils in old *Smyd2*^−/−^ mice, the relevance of which remains to be addressed. Thus, Smyd2 deficient-mice showed largely normal hematopoiesis at steady state.

### *Smyd2* deletion delays MLL-AF9-induced leukemogenesis

**Figure 3:**
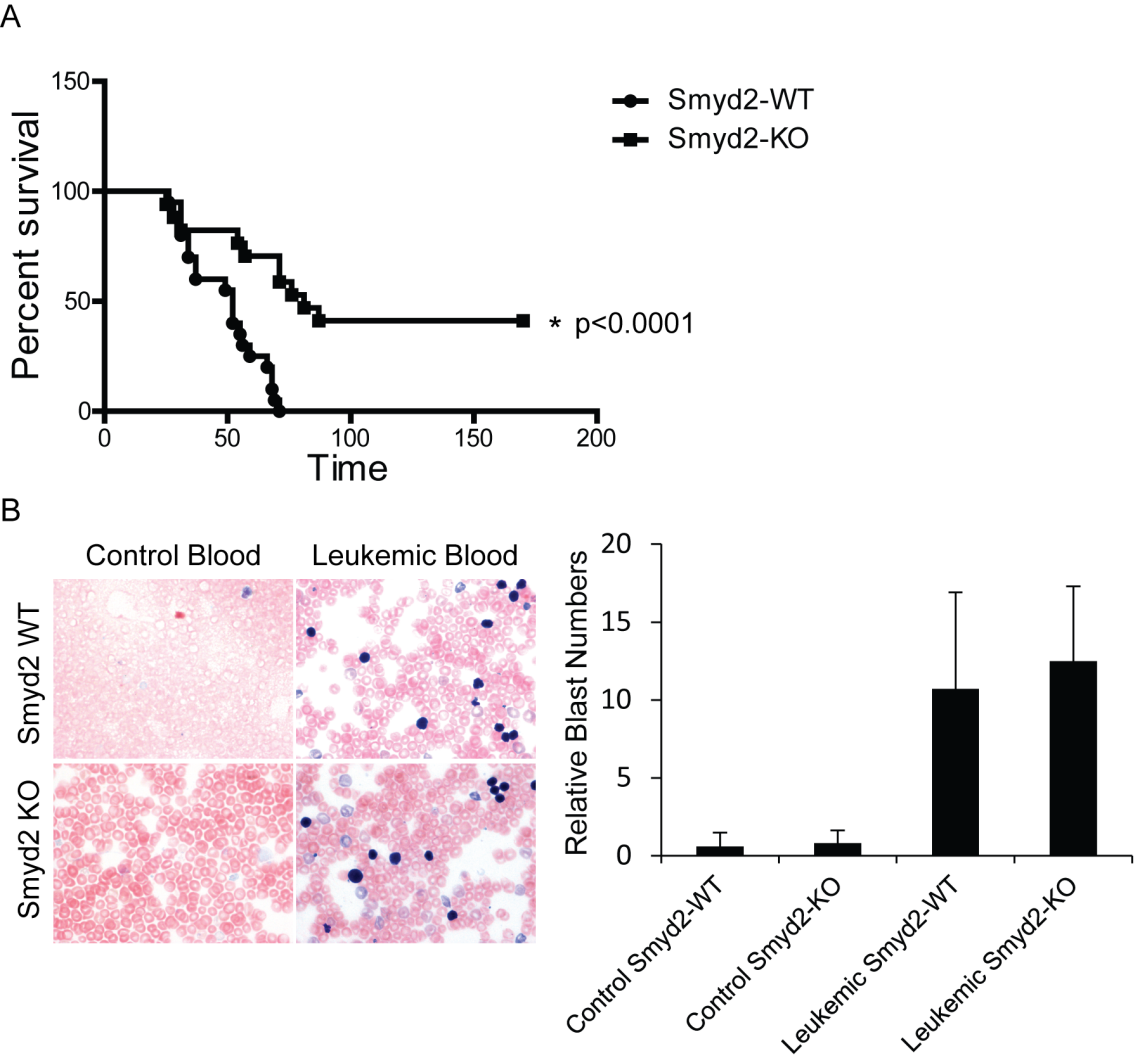
Smyd2 deletion impairs MLL-AF9/NRas^G12D^-driven leukemogenesis **A.** Kaplan Meier curve representing disease-free survival of recipient mice transplanted with MLL-AF9/NRas^G12D^-infected HSPCs derived from *Smyd2*^+/+^ and *Smyd2*^−/−^ donors, as indicated. HSPCs from 6 *Smyd2*^+/+^ and 5 *Smyd2*^−/−^ donors were transferred into 20 and 17 recipients, respectively. Log rank test shows a statistically significant survival advantage in *Smyd2*^−/−^ HSPC reconstituted AMLs (*p* < 0.0001). **B.** Representative peripheral blood smears from control and leukemic recipient mice of *Smyd2*^+/+^ and *Smyd2*^−/−^ genotypes (60X objective). Leukemic recipients have significantly high amount of circulating immature blasts. The samples were stained with May-Grünwald-Giemsa (Left panel). Counting the number of blast cells of 10 regions under the microscope using 60X objective revealed that no significant difference in the numbers of circulating blast cells between the leukemic recipients of *Smyd2*^+/+^ and *Smyd2*^−/−^ progenitor cells (Right panel); *p* = 0.62 (Student's *t*-test).

**Figure 4:**
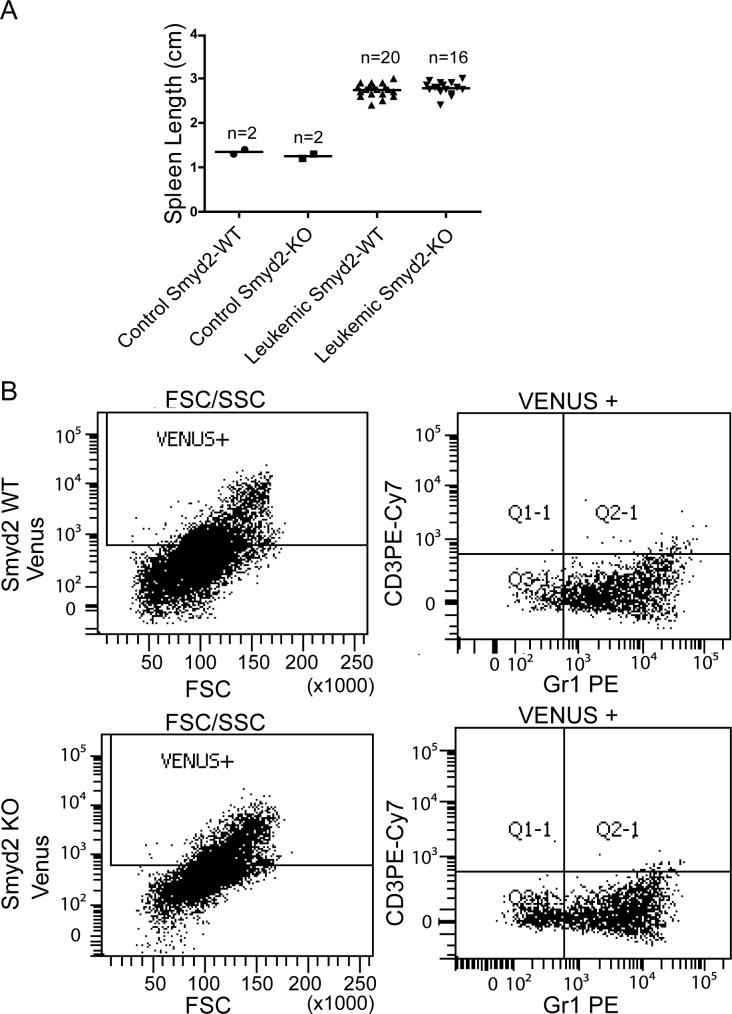
Spleen size and immunophenotypic analysis of MLL-AF9/NRas^G12D^ induced leukemic recipients **A.** Spleen length in control *Smyd2*^+/+^ and *Smyd2*^−/−^ mice (*n* = 2 each) and recipients with leukemia derived from either *Smyd2*^+/+^ or *Smyd2*^−/−^ HSPCs (*n* = 20 and *n* = 16, respectively). No significant difference was observed between the two leukemic genotypes (p=0.4; Student's *t*-test). **B.** Representative immunophenotypic analysis of Venus-positive peripheral blood cells from *Smyd2*^+/+^ and *Smyd2*^−/−^ leukemic recipients: detection of Venus positivity, Forward scatter (FSC), GR1 and CD3 staining were assessed by flow-cytometry, as indicated.

In order to test the role of Smyd2 in AML, we transduced *Smyd2*^−/−^ and *Smyd2*^+/+^ hematopoietic stem and progenitor cells (HSPCs) with retroviruses expressing the MLL-AF9 and NRas^G12D^ oncogenes, and transplanted the infected cells into sub-lethally irradiated syngeneic wild-type recipients. Smyd2 deficiency caused a significant delay and reduced penetrance of AML-associated death (Figure 3A). Upon disease development, however, *Smyd2*^−/−^ and *Smyd2*^+/+^ HSPCs gave rise to undistinguishable AML phenotypes, as judged by either leukemic blast counts in peripheral blood (Figure 3B), spleen size (Figure 4A) or the Gr-1^+^CD3^−^ immunophenotype of MLL-AF9-positive tumor cells (tracked by the associated Venus fluorescent marker), confirming their myeloid identity (Figure 4B).

### Lack of *Smyd2* does not impair clonogenic potential but causes competitive disadvantage in leukemic cells *in vitro*

**Figure 5:**
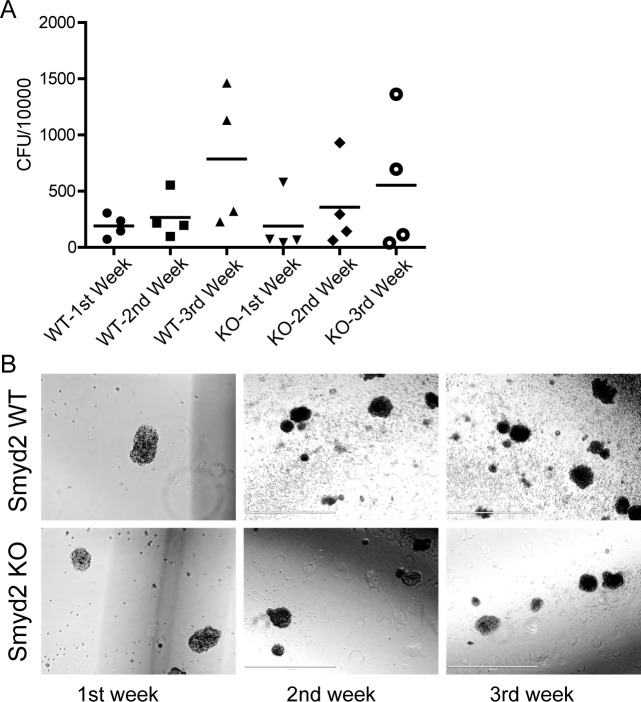
The colony-forming capacity of MLL-AF9/ NRas^G12D^ induced leukemic stem cells is unaffected by the loss of Smyd2 **A.** Colony forming units (CFU) per 10,000 primary leukemic cells sorted from the spleen of leukemic recipients. The cells were plated in methylcellulose supplemented with HSPC cytokines, and serially passaged three times. **B.** Morphology and size of representative blast colonies from each genotype (10X objective, scale bar 1000 μm).

We then compared the growth and colony forming capacity of *Smyd2*^+/+^ and *Smyd2*^−/−^ leukemic cells in semi-solid medium. Although clonogenic capacity was variable among biological samples of the same phenotype, the range was comparable between the two groups (Figure 5A), with comparable colony shape and size (Figure 5B).

**Figure 6:**
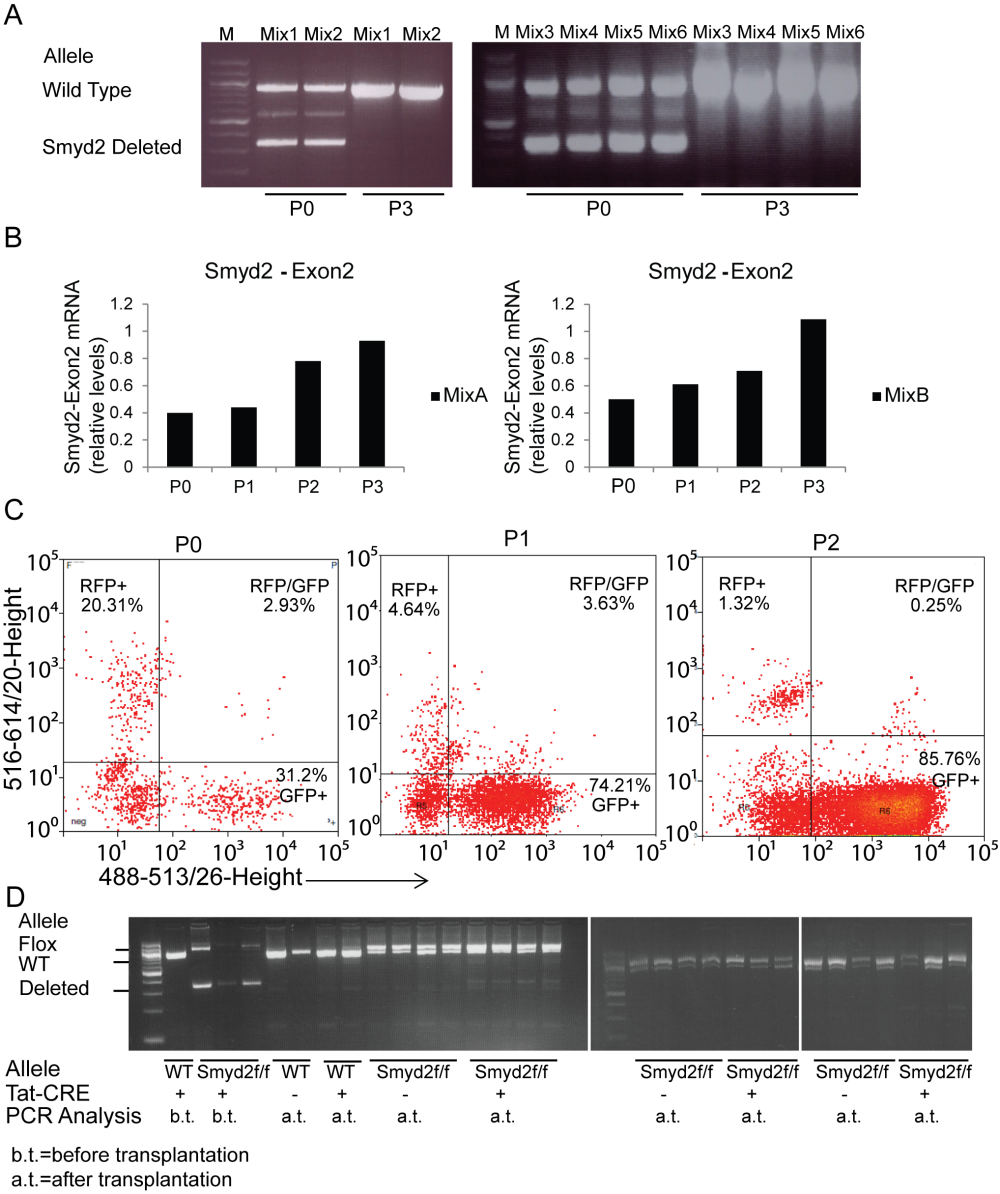
Competitive disadvantage of Smyd2-deleted leukemic cells **A.** *In vitro* competition assay: PCR analysis of wild type and deleted *Smyd2* alleles in cell mixtures. *Smyd2*^+/+^ and *Smyd2*^−/−^ primary leukemic cells were mixed (1:1 ratio) and analyzed at passages 0 and 3. Six independent mixes using 4 *Smyd2*^+/+^ and *Smyd2*^−/−^ biological replicates were used in this assay. Primers that flank *Smyd2* exon2 (the deleted exon in *Smyd2*-null cells) were used for the PCR, allowing simultaneous detection of the wild type and deleted alleles. **B.** Quantitative RT-PCR analysis of Smyd2 exon 2 in two representative mixtures at the indicated passages. The *Tbp* gene was used as a normalizer. The bars show the average of two experimental replicates. **C.** Flow cytometric analysis of the selective disadvantage of *Smyd2*^−/−^ leukemic cells. Wild type and *Smyd2*^−/−^ leukemic cells were infected with pMSCV-GFP and pMSCV-cherry retroviruses, respectively. The experiment was repeated 3 times with different biological replicates, with very similar results: a representative experiment is shown here. **D.** *In vivo* competition assay: primary *Smyd2^flox/flox^* leukemic cells were incubated with either Tat-CRE (+) or vehicle (−), and transplanted into secondary recipients. PCR analysis: DNA samples were analyzed either before transplantation (b.t) or after transplantation and development of secondary leukemia (a.t.) with the same primers as in A. Loss of the *Smyd2*-deleted relative to the non-deleted (Flox) allele is clearly visible in the secondary leukemia derived from Tat-CRE incubated cells, indicating loss of the deleted cells.

To address the competitive fitness of *Smyd2*^+/+^ relative to *Smyd2*^−/−^ leukemic cells, we mixed these cells in 1:1 proportions, and serially passaged the resulting mixtures in liquid culture. The relative amounts of cells of each genotype were assessed by monitoring the proportion of wild type and knockout *Smyd2* alleles by PCR analysis (Figure 6A): Smyd2-deleted cells were reproducibly eliminated, wild-type leukemic cells taking over the cultures at the third passage. As the same result was obtained in all the mixtures, the loss of the mutant allele was thus unlikely to stem from stochastic clonal variations, but rather from a selective disadvantage of *Smyd2*^−/−^ leukemic cells upon serial replating. Quantitative RT-PCR analysis of the wild-type *Smyd2* mRNA in 2 mixed populations showed a progressive increase with ultimate doubling at passage 3, consistent with the full takeover of the cultures by wild-type cells (Figure 6B). Finally, wild-type leukemic cells infected with a pMSCV-GFP vector were mixed with *Smyd2*^−/−^ leukemic cells infected with pMSCV-Cherry and serially passaged, revealing a gradual decrease in Cherry- and increase in GFP-positive cells (Figure 6C).

### *Smyd2*-null leukemic cells show competitive disadvantage *in vivo*

We next aimed to test the maintenance of *Smyd2*-deleted leukemic cells *in vivo*. Primary leukemia were generated by introducing MLL-AF9 and NRas^G12D^ in either *Smyd2^flox/flox^* or *Smyd2^wt/wt^* HSPCs. Leukemic cells collected from the spleens of recipient mice were incubated *in vitro* with a recombinant Tat-CRE recombinase [37]. PCR amplification, performed to test the level of *Smyd2* deletion, revealed incomplete deletion of the *Smyd2^flox^* allele (the percentage of the deleted allele was 61, 82, and 75 respectively for the three *Smyd2^flox/flox^* donors), resulting in heterogeneous populations containing both Smyd2 deleted and undeleted cells. Each Tat-CRE- and vehicle-treated leukemia sample was transplanted into 4 recipients. PCR analysis of secondary leukemia samples showed elimination of the mutant allele (Figure 6D), indicating a competitive disadvantage of Smyd2-deleted cells *in vivo*.

**Figure 7:**
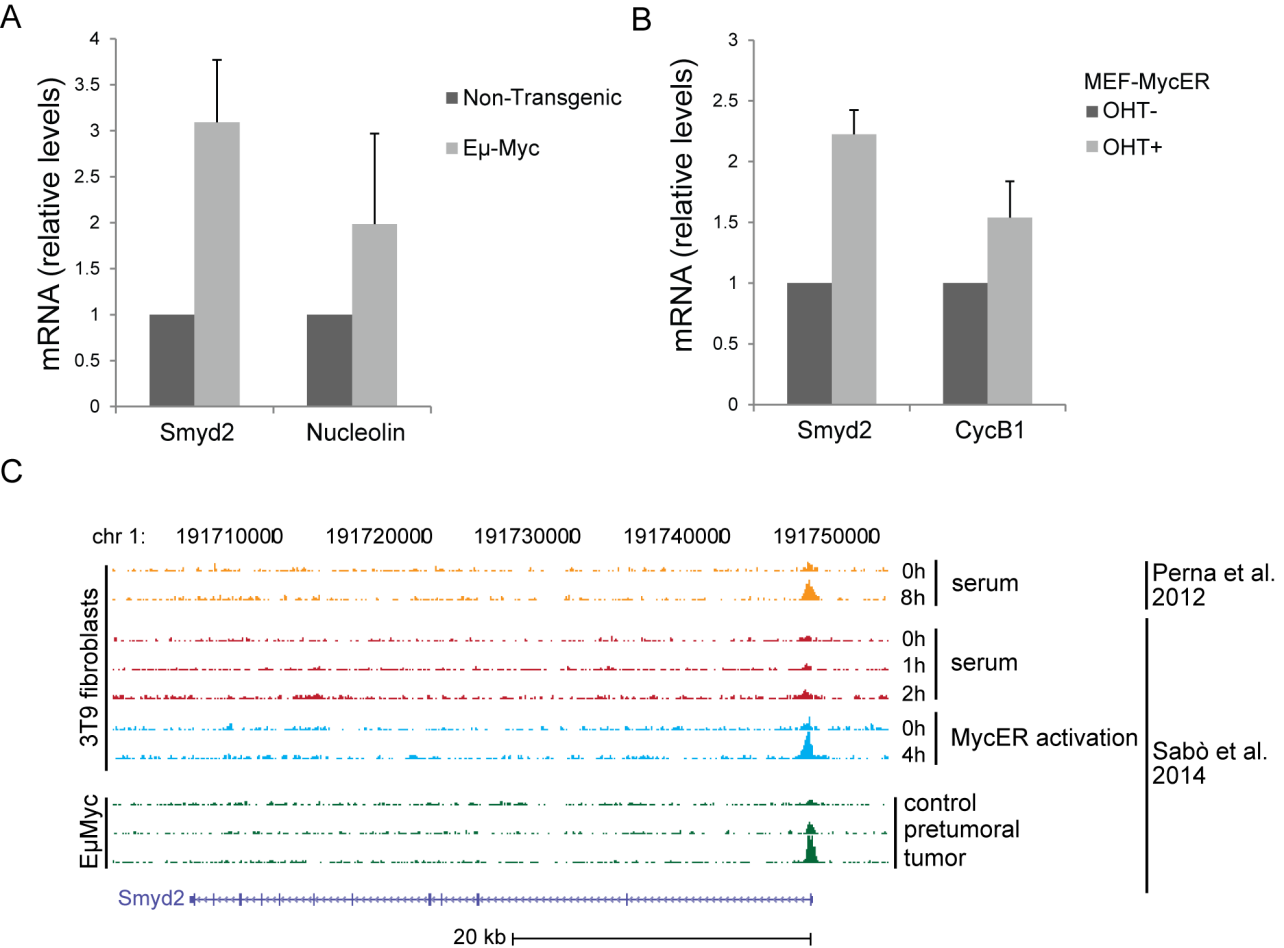
Smyd2 is a direct Myc target Quantitative RT-PCR analysis of the *Smyd2* mRNA in splenic B-cells of Eμ-*myc* and non-transgenic mice **A.** and upon hydroxy-tamoxifen (OHT)-induced MycER activation in mouse embryo fibroblasts (MEF-MycER) **B.** *Nucleolin* and *Cyclin B1* (*CycB1*) are known Myc targets, used as positive controls. Averages values and error bars are from three biological replicates. *Tbp* was used as the house-keeping gene to calculate relative mRNA levels. **C.** Myc ChIP-Seq profiles at the *Smyd2* locus in 3T9 fibroblasts and B-cell samples, as indicated [8, 9].

### The *Smyd2* gene is a direct Myc target

**Figure 8:**
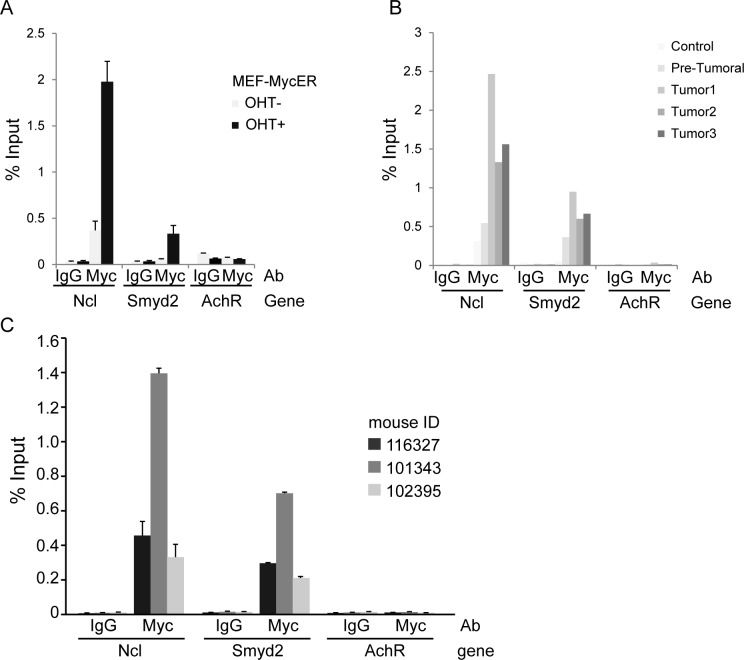
ChIP-qPCR analysis confirms binding of Myc to the *Smyd2* promoter **A.** Myc-ER fibroblasts; **B.** Non-transgenic, pre-tumoral and tumoral Eμ-*myc* transgenic B-cells. *Ncl* (*nucleolin*) and *AchR* (*acetylcholine receptor*) were used as positive and negative controls, respectively. **C.** ChIP-qPCR analysis of Myc enrichment on the *Smyd2* promoter in splenic cell samples obtained from three terminally sick MLL-AF9 leukemic recipients. The bars represent the average of three experimental replicates in Fig. 8A and C, and two experimental replicates in Fig. 8B.

Transcription factors, such as Myb and c-Myc, mediate the regulatory network exerted by MLL fusion proteins. In the same leukemia model used here, in particular, MLL-AF9 bound to the c-*myc* promoter and induced expression of the gene [7]. In our previous RNA profiling datasets, *Smyd2* classified as a Myc-dependent serum response (MDSR) gene in mouse fibroblasts [8] and was induced during lymphoma development in Eμ-*myc* mice [9], the latter validated here by RT-PCR (Figure 7A). Albeit not classified as significant in our RNA-seq data [9], *Smyd2* also showed activation following ectopic MycER activation in fibroblasts (Figure 7B). In all of the above models, ChIP-Seq profiles showed that Myc bound to the *Smyd2* promoter, indicating that *Smyd2* activation by Myc is direct (Figure 7C). Chip-PCR data confirmed binding of Myc to the *Smyd2* promoter in MEF-MycER and Eμ-*myc* B-cells (Figure 8A, 8B). Using the same assay, we showed that the *Smyd2* promoter was directly targeted by Myc in MLL-AF9 leukemic cells from three independent tumors (Figure 8C). Altogether, our results indicate that *Smyd2* is activated by Myc downstream of MLL-AF9 and is a critical mediator of the oncogenic signal.

### Smyd2 is dispensable for Myc-induced lymphomagenesis

**Figure 9:**
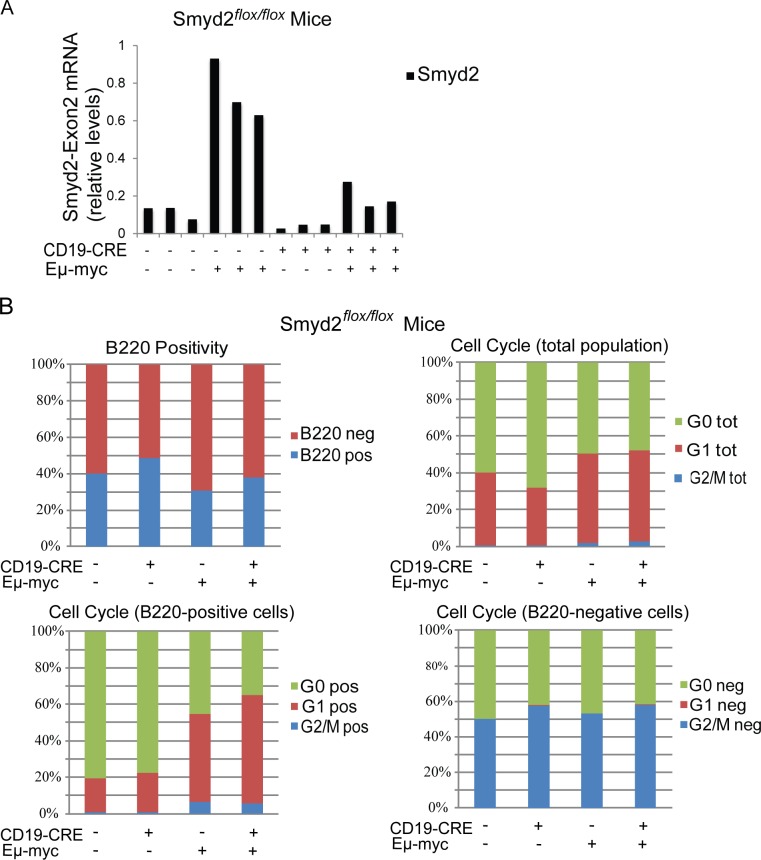
Smyd2 deletion does not affect pre-tumoral B cell expansion and cell cycle distribution in Eμ-*myc* transgenic mice **A.** Quantitative RT-PCR analysis of Smyd2 exon2 in the B cells isolated from mice of the indicated genotypes (*n* = 3 each). Relative *Smyd2* mRNA levels were calculated using *Tbp* as the housekeeper gene. The bars represent the average of two experimental replicates. **B.** Multiparameter FACS analysis of peripheral blood cells. Cells were stained with B220-PE or B220-PE and Ki67-Alexa 488 just before FACS acquisition. Cell Cycle distributions based on Ki67 staining are shown for the total, the B220-positive and the B220-negative cell populations. Six biological replicates were included in the analysis for each genotype, with negligible standard deviations (not included).

**Figure 10:**
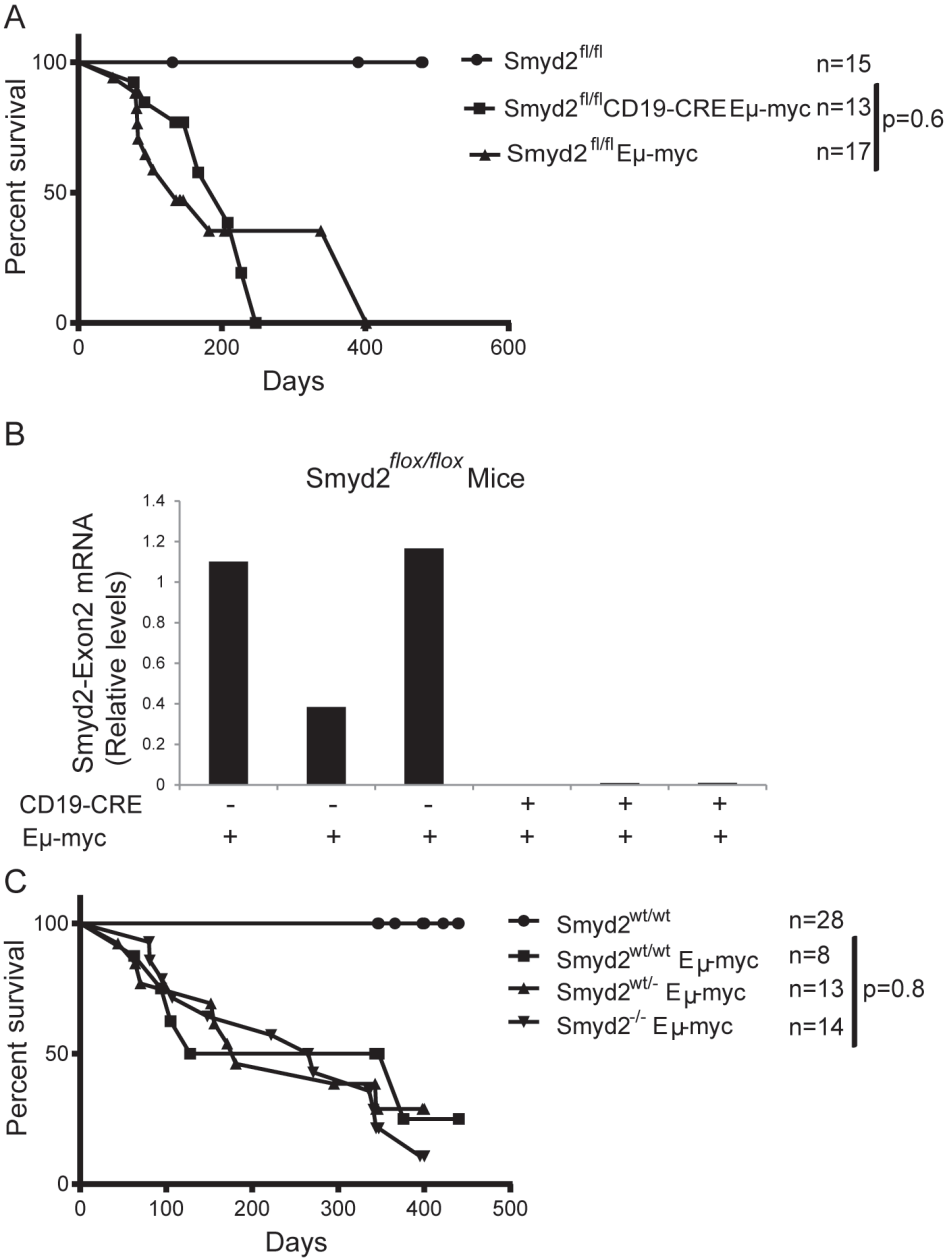
Eμ-*myc* induced lymphoma formation was not affected by Smyd2 deletion **A.** Disease-free survival curves for control (*Smyd2^flox/flox^*), Eμ-*myc Smyd2^flox/flox^* and Eμ-*myc Smyd2^flox/flox^ CD19-CRE* mice. **B.** Quantitative RT-PCR analysis of Smyd2 exon2 shows complete elimination of *Smyd2* by *CD19-CRE* in lymphoma samples. The bars represent the average of two experimental replicates. **C.** Survival curves for control (*Smyd2^wt/wt^*) and Eμ-*myc* transgenic mice of the indicated Smyd2 genotypes. P-values were calculated using Log-rank (Mantel-Cox) test.

To address the role of Smyd2 in Myc-induced lymphoma formation, Eμ-*myc* transgenic mice [38] were bred with *Smyd2^flox/flox^ CD19-CRE* mice. Conditional deletion of *Smyd2* was tested by quantitative RT-PCR on RNA samples isolated from the pre-tumoral B cells of 6-8 weeks old animals (Figure 9A). Flow-cytometry with B220 and Ki67 staining showed that the cell cycle distribution of B cells in the blood of young (pre-tumoral) mice was not affected by the loss of Smyd2 (Figure 9B). Consistent with this result, Eμ-*myc Smyd2^flox/flox^* mice with or without *CD19-CRE* showed no significant difference in disease onset (Figure 10A). The absence of *Smyd2* in lymphomas arising with *CD19-CRE* was confirmed at the mRNA level (Figure 10B): it is noteworthy here that lymphomas are generally monoclonal, explaining the full loss of *Smyd2*, while mixed pre-tumoral populations still showed residual expression (Figure 9A). Finally, germ-line deleted Eμ-*myc Smyd2*^−/−^ animals and Eμ-*myc Smyd2*^+/+^ controls showed comparable lymphoma onset (Figure 10C). Thus, *Smyd2* deletion did not affect Myc-induced lymphomagenesis. We note that, albeit not significantly deregulated during lymphomagenesis, *Smyd3* is expressed in mouse B-cells [9]: whether combined loss of *Smyd2* and *Smyd3* may affect lymphomagenesis - or may further impair AML progression - remains to be addressed.

## DISCUSSION

Our study addressed the role of Smyd2 methyltransferase in normal mammalian development, hematopoiesis, and MLL-AF9 induced leukemic transformation. Smyd2 is one of the five MYND domain containing SET-proteins, with demonstrated methyltransferase activity. Unlike *Smyd1*, which is expressed in heart and muscle in mouse and human [11, 39, 40], *Smyd2* and -*3* are expressed in a broad range of tissues [14]. While Smyd1 is required for mouse development, knockout animals dying at embryonic day 10.5 [11], we show here that germ-line deletion of either *Smyd2* or *Smyd3*, or the two together, has no obvious effect on mouse development, fertility or lifespan.

A series of studies pointed to the potential involvement of Smyd2 and Smyd3 in various human cancers [16, 17, 22–24, 41–44]. In our hands, mining of the Oncomine database revealed that *SMYD2* transcription is significantly elevated in patients with bladder cancer, colon adenoma and colorectal carcinoma, hepatocellular carcinoma, prostate carcinoma, hereditary clear cell renal cell carcinoma, and chronic lymphocytic leukemia (Oncomine™; Compendia Bioscience, Ann Arbor, MI). SMYD2 was also reported to methylate several cancer-associated substrates, including p53, RB and ERα [26, 30, 31]. Nevertheless, the involvement of Smyd2 in different types of tumors remained to be evaluated experimentally. Moreover, the role of Smyd2 in normal development had never been genetically addressed.

As enforced Smyd2 expression has been associated with elevated self-renewal of hematopoietic progenitors [7], we investigated the number of total bone marrow, lineage-depleted cells, and differentiated circulating blood cells in *Smyd2* KO mice. According to our results, loss of Smyd2 did not appreciably alter normal hematopoiesis, although more detailed analyses will be required to address its possible roles under stress conditions.

Our data show that Smyd2 is critical for leukemogenesis induced by the MLL-AF9 oncogene, Smyd2 knockout primary leukemic cells showing marked competitive disadvantages over their wild-type counterparts either *in vitro* or *in vivo*. We demonstrate that the *Smyd2* gene is a direct target of Myc, which itself was proposed to be important for stemness in leukemic cells [45]. Moreover, *Smyd2* is directly targeted by Myc in fibroblasts and B-cells, including normal and Myc-transformed cells. The c-myc gene itself is a direct transcription target of MLL-AF9, as well as of the Myb transcription factor, which may mediate oncogene addiction in AML [7]. Altogether, these observations point to a transcriptional regulatory cascade in leukemic cells, with MLL-AF9 and Myb activating *c-myc*, leading to Myc accumulation and activation of *Smyd2*.

The role of Smyd2 downstream of Myc must be dependent on cellular context, as *Smyd2* deletion did not affect lymphomagenesis in Eμ-*myc* transgenic mice. Interestingly, while MLL-AF9-induced leukemias depend on a subset of self-renewing stem cells [46], this does not appear to be the case in Eμ-*myc* lymphomas [47]. We speculate that Smyd2 is part of a Myc-controlled self-renewal network downstream of MLL-AF9.

## MATERIALS AND METHODS

### Mouse breedings and handling

C57Bl/6J mouse strains carrying the *Smyd2^tm1a(KOMP)Wtsi^*and *Smyd3^tm1a(KOMP)Wtsi^* alleles, respectively, were obtained from the European Conditional Mouse Mutagenesis Consortium (Eucomm). These alleles include *LoxP* elements flanking exon 2 of the *Smyd2* and *Smyd3*, respectively, and an *FRT*-flanked selection cassette containing the *Engrailed-1* splice acceptor (sA), *β-Gal* and neomycin selection elements. Animals from both strains were crossed with *FlpE* recombinase transgenic mice [48] in order to eliminate the selection cassette, resulting in the *Smyd2^flox^* or *Smyd3^flox^* allele. Homozygous *Smyd2^fl/fl^* and *Smyd3^fl/fl^* mice were viable and fertile, and showed no abnormalities compared to fl/wt or wt/wt counterparts. In order to delete *Smyd2*, or *Smyd3*, from the germ line, homozygous flox/flox mice were crossed with the *Deleter* strain [49]. Heterozygous *Smyd2* or *Smyd3*-targeted mice, obtained from the *Smyd2^fl/fl^*-*Deleter* or *Smyd3^fl/fl^*- *Deleter* crosses, were then bred with wild type mice in order to obtain *Smyd2^+/−^* or *Smyd3^+/−^* free of the *Deleter*-*Cre* allele. The heterozygous mice were then intercrossed to generate *Smyd2*^−/−^ and *Smyd3*^−/−^ mice, which were subsequently used to obtain double knockout animals. Mice were maintained on a C57Bl/6 background. To target Smyd2 in lymphomas, *Smyd2^fl/fl^* mice were first bred with C57Bl/6J *CD19-CRE* transgenic mice [50], and *Smyd2^fl/+^ CD19-CRE* mice then crossed to obtain *Smyd2^fl/fl^ CD19-CRE* animals. The latter were bred with C57Bl/6J Eμ-*myc* transgenic mice. The resulting Eμ-*myc Smyd2^fl/fl^* and Eμ-*myc Smyd2^fl/fl^*, *CD19-CRE* animals were monitored twice a week for lymphoma development by lymph node palpation. Alternatively, C57Bl/6J Eμ-*myc* transgenic mice were serially bred with germ line deleted *Smyd2*^−/−^ mice. The resulting *Smyd2* wild type, heterozygous and knock-out Eμ-*myc* transgenic littermates were monitored for lymphoma development as described before. The PCR primers used for genotyping all strains are listed in supplementary Table 2.

Experiments involving animals have been done in accordance with the Italian Laws (D.lgs. 26/2014), which enforces Dir. 2010/63/EU (Directive 2010/63/EU of the European Parliament and of the Council of 22 September 2010 on the protection of animals used for scientific purposes).

### Generation of AML

AML was modeled as described [7]. Lineage-depleted hematopoietic stem and progenitor cells (HSPCs), were isolated from total bone marrow of *Smyd2*^−/−^ and *Smyd2*^+/+^ mice with the Lineage Cell Depletion Kit, mouse (cat. no. 130-090-858, Milteny Biotech). The cells were cultured overnight in HSC specific medium (RPMI, 10% HSC qualified FBS, 50 μg/ml SCF, 10 ng/ml IL3, 10 ng/ml IL6) before being infected with a 1:1 mix of pMSCV-MLL-AF9-IRES-Venus and p-MSCV-Luci-IRES-mNRas^G12D^ retroviruses [7]. Two days after infection, HSPCs were transplanted by tail vein injection into sub-lethally irradiated (7 grays) syngeneic recipients (1.5×10^5^ cells/mouse). Leukemia onset was diagnosed by the presence of the myeloblastoid cells in blood smears detected by May-Grünwald-Giemsa staining, peripheral blood cell counts inBeckman-Coulter automated blood cell counter, and observation of enlarged spleen by palpation. The percentages of leukemic cells were determined by flow cytometric detection of Venus positivity. Flow-cytometric analysis of Gr-1-PE (BD Biosciences Pharmingen, cat. no. 561084, 1/100 dilution) and CD3- PE Cy7 (BD Biosciences Pharmingen, cat. no. 561100, 1/200 dilution) staining was used to confirm myeloid origin of the Venus positive tumor cells. Statistical evaluation of survival was performed by the log-rank (Mantel-Cox) test for comparison of the Kaplan-Meier event time format.

### Colony forming assay

Leukemic spleen cells were sorted for Venus (MLL-AF9 oncogene) positivity, and 10.000 cells from 4 biological replicates of each genotype, obtained from recipients transformed with different HSPC donors, were seeded in methyl cellulose (MethoCult M3234, StemCell Technologies) supplemented with HSPC cytokines (50 μg/ml SCF, 10 ng/ml IL3, 10 ng/ml IL6). The colonies were observed and counted for three weeks, with weekly passages.

### Competition assays

For *in vitro* competition assays, *Smyd2*^+/+^ and *Smyd2*^−/−^ leukemic cells, obtained from sorting the Venus positive spleen cells from terminally sick mice, were mixed in 1:1 proportion and seeded in HSC medium. The DNA preparations obtained from each passage were subjected to a PCR analysis (28 cycles) using a set of primers which amplify wild type, flox and deleted alleles (Smyd2_flox-F and Smyd2_flox-R) (Supplementary Table 2). *Smyd2* RNA levels in wild type/Smyd2-null leukemia mixtures were monitored by quantitative RT-PCR with a primer set that amplifies exon 2 (Supplementary Table 2).

For *in vivo* competition assays, MLL-AF9 leukemias were generated from the HSPCs of 3 *Smyd2^fl/fl^* and 2 *Smyd2^w/w^* donors. Leukemic spleen cells were isolated as above and incubated with custom made recombinant Tat-CRE recombinase (100 μM, for 3 hours) [37]. Tat-CRE and vehicle treated leukemia samples were separately transplanted into 4 recipients each, as described above (0.9×10^6^ cells per recipient). Recipient animals were irradiated (5 gray) 24 hours before tail vein injection. Secondary leukemia occurrence was observed from 17 to 37 days after transplantation, monitoring general physical health, blood cell counts and enlarged spleen, as described above. *Smyd2* alleles were analyzed by PCR, using the primers described above (Smyd2_flox-F and Smyd2_flox-R) on the DNA samples of Venus-sorted spleen cells.

### Primary cells and cell lines

Bone marrow cells were isolated from tibias and femurs of the mice, stained with trypan blue, and counted in a haemocytometer. Lineage-depleted cells (lin-) were obtained from total bone marrow cells using the Lineage Cell Depletion Kit, mouse (cat. no. 130-090-858, Milteny Biotech) and counted as above.

For pre-tumoral analysis, blood samples from 6-8 weeks old Eμ-*myc* transgenic mice with no infiltration of peripheral lymph nodes were used. Isolation and processing of control, pretumoral and tumor primary B-cells for ChIP assays were performed as described [9]. The 3T9-MycER fibroblast cells used in ChIP assay were described [9]. Fibroblasts were grown in DMEM medium supplemented with 10% serum, penicillin/streptomycin, 2 mM L-Gln and 1% β-mercaptoethanol in low-oxygen conditions (3%).

The antibodies used in multiparameter flow cytometry to characterize pre-tumoral B cells are; B220-PE (1/200 dilution, cat. no. 553081, BD Pharmingen), and Ki67-Alexa 488 (1/25 dilution, cat. no. 561165, BD Pharmingen). For Ki67 staining; the B220-PE stained cells were fixed in formaldehyde and permeabilized using the permeabilization solution from the Ki67 staining kit (BD Biosciences Pharmingen, cat. no. 558616) for 30 minutes, and incubated with the Ki67 antibody for 1 hour. 1/10000 diluted Hoechst solution was used to counter-stain the cells (cat. no. 33342, Life Technologies).

### Chromatin immunoprecipitation

ChIP assay was performed as described [9]. For the leukemia ChIP analysis, 30 million spleen cells from three wild type leukemic recipients from different donors were seeded in HSC medium supplemented with cytokines 48 hours before formaldehyde fixation. Myc N262 (Santa Cruz, sc-764) and rabbit IgG (Santa Cruz, sc-2027) antibodies were used for ChIP. The primers used for Q-PCR analysis following ChIP are listed in Supplementary Table 2.

### Analysis of protein identity

Uniprot Alignment Tool was used to determine the percent identities of full-length proteins, or the SET and MYND domains separately [51], as shown in Table 1. The accession numbers used were Q8R5A0 (mouse Smyd1), P97443 (mouse Smyd2), Q9CWR2 (mouse Smyd3), Q8BTK5 (mouse Smyd4), Q3TYX3 (mouse Smyd5).

## SUPPLEMENTARY MATERIAL TABLES




